# Ventilatory Efficiency During Exercise in CTEPH and PAH Pre‐ and Post‐Treatment

**DOI:** 10.1002/pul2.70334

**Published:** 2026-06-04

**Authors:** Esther I. Schwarz, Lara Benning, Zoe Bousraou, Julian Müller, Mona Lichtblau, Isabelle Opitz, Silvia Ulrich

**Affiliations:** ^1^ Department of Pulmonology University Hospital Zurich Zurich Switzerland; ^2^ Faculty of Medicine University of Zurich Zurich Switzerland; ^3^ Department of Thoracic Surgery University Hospital Zurich Zurich Switzerland

**Keywords:** cardiopulmonary exercise testing, chronic thrombo‐embolic pulmonary hypertension, pulmonary arterial hypertension, pulmonary vascular disease, ventilatory efficiency

## Abstract

Ventilatory inefficiency, assessed by the VE/VCO_2_ slope during cardiopulmonary exercise testing (CPET), is a key physiological marker. However, differences in ventilatory efficiency between chronic thromboembolic pulmonary hypertension (CTEPH) and pulmonary arterial hypertension (PAH), as well as their response to treatment, remain incompletely characterized. We retrospectively analyzed CPET data from 92 patients with CTEPH and 91 with PAH. Ventilatory efficiency (VE/VCO_2_ slope) and peak oxygen uptake (peak VO_2_) were compared at baseline and in treatment‐naïve subgroups. Predictors of ventilatory efficiency were assessed using regression analyses. Longitudinal changes following treatment were evaluated in patients with serial CPETs. At baseline, ventilatory efficiency was significantly worse in CTEPH than in PAH (VE/VCO_2_ slope median (IQR) 39.8 (34.3–47.5) versus 35.9 (31–41.5), *p* < 0.001), despite less severe hemodynamic impairment. This difference persisted in treatment‐naïve patients. In both groups, pulmonary vascular resistance, mean pulmonary artery pressure, and peak VO_2_ were independent predictors of the VE/VCO_2_ slope. Following treatment, ventilatory efficiency improved significantly in CTEPH (ΔVE/VCO_2_ slope −8, 95% CI − 13.3 to −4.2, *p* < 0.001), accompanied by an increase in peak VO_2_. Improvements were most pronounced after pulmonary endarterectomy. Patients with PAH showed only modest changes after initiation of targeted therapy. Ventilatory inefficiency is more pronounced in CTEPH than in PAH despite less severe resting hemodynamics. Treatment, particularly pulmonary endarterectomy, leads to substantial improvement in ventilatory efficiency in CTEPH, whereas changes in PAH are less pronounced. CPET‐derived ventilatory efficiency may serve as a useful non‐invasive marker of treatment response in pulmonary hypertension, particularly in CTEPH.

Dyspnea and exercise intolerance are hallmark symptoms of pulmonary vascular diseases, including pulmonary arterial hypertension (PAH, WHO Group 1) and chronic thromboembolic pulmonary hypertension (CTEPH, WHO Group 4). Treatment of PAH is aimed at alleviating dyspnea, enhancing exercise capacity, improving quality of life, slowing disease progression, and reducing mortality [1]. Therapeutic strategies involve the use of drugs that act on three main pathways: the endothelin pathway (endothelin receptor antagonists), the nitric oxide pathway (phosphodiesterase‐5 inhibitors and soluble guanylate cyclase stimulators), and the prostacyclin pathway (prostacyclin analogs and prostacyclin receptor agonizts). Additionally, sotatercept (a fusion protein targeting the TGF‐β superfamily signaling pathway) has emerged as a promising therapy that restores the balance between pro‐proliferative and anti‐proliferative signals in the pulmonary vasculature [2, 3]. Treatment is tailored based on disease severity, risk stratification, and response to therapy. The cornerstone of treatment in CTEPH is pulmonary endarterectomy (PEA), a surgical procedure with the potential to normalize pulmonary hemodynamics by removing organized thromboembolic material from the pulmonary arteries. For patients with segmental and subsegmental CTEPH in whom the thromboembolic lesions are not surgically accessible or who are inoperable, balloon pulmonary angioplasty (BPA) is an option. Targeted medical therapy is approved for patients with distal CTEPH or persistent precapillary pulmonary hypertension after PEA [4, 5].

Cardiopulmonary exercise testing (CPET) plays an important role in the assessment and management of pulmonary vascular disease. A typical CPET pattern in PAH includes an elevated ventilatory equivalent for carbon dioxide (VE/VCO_2_ slope), a reduced oxygen pulse, decreased end‐tidal CO_2_, and a diminished peak oxygen uptake (VO_2_ peak). According to current PAH guidelines, CPET values such as VO_2_ peak and VE/VCO_2_ slope contribute to distinguishing low‐ (VE/VCO_2_ slope < 36), intermediate‐ (VE/VCO_2_ slope 36–44), and high‐risk patients (VE/VCO_2_ slope > 44) at diagnosis, guiding both prognosis and therapeutic decisions [4]. CPET is recommended at baseline and may be considered during follow‐up (e.g., every 6 months or in response to clinical changes) [4].

Ventilatory efficiency during exercise is one of the most important determinants of exercise capacity and tolerance. In CPET, ventilatory efficiency is assessed via the VE/VCO_2_ slope and the VE/VCO_2_ ratio at the anaerobic threshold (VE/VCO_2__AT). The VE/VCO_2_ slope is the slope of the linear relationship between ventilation and carbon dioxide output during exercise [6]. A VE/VCO_2_ slope less than 30 is widely accepted as normal in healthy adults. Reference values for the VE/VCO_2_ slope indicate a median of 22 with a 2.5th and 97.5th percentiles of 19.1 and 30.7 associated with sex and age [7]. Ventilatory efficiency during exercise declines with age [8]. Higher slopes (e.g., > 35) are associated with impaired ventilatory efficiency and are not only indicative of PAH and CTEPH but also of conditions such as heart failure, chronic obstructive pulmonary disease (COPD) or interstitial lung disease (ILD). Ventilatory efficiency has been shown to play a role in assessing the prognosis of these cardiopulmonary diseases [9, 10, 11, 12].

Despite the recognized importance of ventilatory efficiency, as reflected by the VE/VCO_2_ slope, in risk stratification in pulmonary hypertension guidelines, there is a notable paucity of data on how this parameter responds to treatment in both PAH and CTEPH, as well as limited evidence directly comparing ventilatory efficiency between these two forms of pulmonary vascular disease [13].

This study aimed to test the hypothesis that ventilatory efficiency is more severely impaired in patients with CTEPH compared to those with PAH, and to explore whether improvements in ventilatory efficiency following treatment are more pronounced in CTEPH and whether ventilatory efficiency correlates with measures of disease severity such as mean pulmonary artery pressure (mPAP) or pulmonary vascular resistance (PVR).

## 1 Methods

### 1.1 Study Design and Patient Population

This was a retrospective longitudinal observational study conducted at the reference center for pulmonary hypertension at the University Hospital Zurich in Switzerland, aimed at comparing ventilatory efficiency (VE/VCO_2_ slope) obtained from CPET in patients with CTEPH and PAH and at assessing changes in the VE/VCO_2_ slope following initiation and escalation to maximal targeted therapy (drug therapy with pulmonary vasodilators, BPA, PEA). Adult patients (≥ 18 years) with a confirmed diagnosis of either PAH (idiopathic, hereditary, drug‐induced, associated with HIV or associated with connective tissue disease) or CTEPH were eligible for inclusion. Diagnosis was established according to the European Society of Cardiology (ESC) and European Respiratory Society (ERS) guidelines for pulmonary hypertension at that time [4, 14]. Patients with congenital heart disease, co‐existent moderate to severe interstitial or obstructive lung disease or heart failure with preserved or reduced ejection fraction were excluded.

Patients were eligible if they underwent at least one clinically indicated cycle ergometer CPET using an incremental protocol between 2014 and 2024. Ideally, three CPETs were performed: one at baseline (prior to or at the initiation of specific therapy) and two during follow‐up, with intervals between tests ranging from a minimum of 6 months to a maximum of 36 months. All participants had been referred to and treated at the reference center and had provided written informed consent for the use of their coded clinical data for research purposes. The study was approved by the ethics committee of the Kanton of Zurich (KEK‐Nr. 2025‐02640) and conducted in accordance with the Declaration of Helsinki.

### 1.2 Outcomes of Interest

The main outcomes of interest were the VE/CO_2_ slope at baseline and its change in response to therapy. Other outcomes of interest were VE/VCO_2__AT and its change in response to treatment, the association of the VE/VCO_2_ slope and VE/VCO_2_ with peak VO_2_, mPAP, PVR, dead space fraction of the tidal volume (VD/VT), resting arterial partial pressure of CO_2_ (paCO_2_) O_2_ (paO_2_), and single‐breath diffusion capacity for carbon monoxide (DL_CO_). An additional outcome was the change in risk stratification according to VE/VCO_2_ slope values according to a 4‐class and a _3_^−^class system used in heart failure [15, 16].

### 1.3 CPET Protocol and Arterial Blood Gas Analysis

Patients underwent incremental cycle‐ergometer CPETs and were connected to the flow sensor of a metabolic unit via a mouthpiece (Ergostick; Geratherm Medical, Geschwenda, Germany), which was calibrated before each test and measured respiratory gas exchange on a breath‐by‐breath basis (then averaged over 30 s). Patients performed an incremental ramp protocol (same protocol for all three CPETs, 10/15/20 Watt steps per minute to reach an approximate test duration of 10–12 min) and were instructed to cycle until exhaustion (55–65 rounds per minute). Arterial blood gases were taken via radial artery puncture at rest and at peak‐exercise (ABL90 FLEX; Radiometer, Thalwil, Switzerland).

### 1.4 Statistics

Continuous data are presented as median (interquartile range), categorial data as quantity (percentage). Data were tested for normal distribution by Shapiro–Wilk test and quartile–quantile plot. Since data were non‐normally distributed, the Mann–Whitney *U* test was used for the comparison of continuous variables between CTEPH and PAH. The Chi^2^ test was used for the comparison of nominal variables. The Wilcoxon signed‐rank test with subsequent Bonferroni correction was used for within‐group comparison between baseline and follow‐up measurements. Linear regression models were used to identify predictors of the VE/VCO_2_ slope. When residuals were non‐normally distributed, generalized linear models with a gamma distribution were applied; when residuals were heteroscedastic, ordinary least squares regression with robust standard errors was used. Regression models were adjusted for potential clinically relevant confounders. Sample size estimation indicated that 23 patients with CTEPH would be required to detect a treatment effect on the VE/VCO_2_ slope of 3.4 units, assuming a standard deviation of 5.5, with 80% power and a two‐sided significance level of 0.05. This calculation was based on previously reported changes in VE/VCO_2_ slope in CTEPH patients undergoing BPA [17]. However, as CTEPH patients are a heterogeneous group with common comorbidities such as obesity affecting exercise capacity and eventually ventilatory efficiency, the aimed sample size for CTEPH was 30. Based on a small single drug study (sotatercept) reporting the change in VE/VCO_2_ slope in PAH, a paired‐sample size estimation indicated that approximately 24 patients are needed to detect a similar change in VE/VCO_2_ slope with 80% power at a two‐sided *α* = 0.05. Missing data were treated as unavailable; no imputation was performed. RStudio (version 2025.5.0.496, Posit PBC) was used for conducting statistical analyses and design graphs. Where applicable, analyses adhered to the STROBE (Strengthening the Reporting of Observational Studies in Epidemiology) guidelines.

## 2 Results

### 2.1 Patient Population

**Figure 1:**
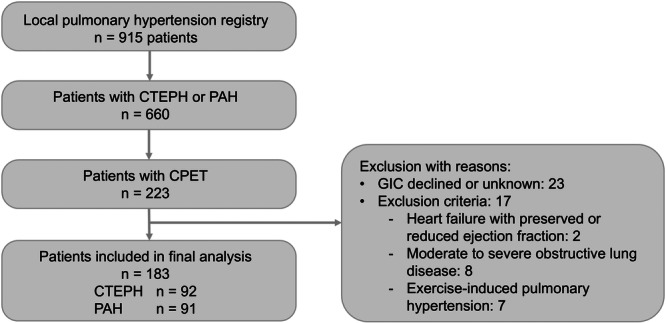
Patient flow. CPET, cardiopulmonary exercise testing; CTEPH, chronic thromboembolic pulmonary hypertension; PAH, pulmonary arterial hypertension.

Ninety‐two patients with CTEPH and 91 patients with PAH fulfilled the eligibility criteria. Eighty‐four patients underwent three repeated CPETs, including a baseline test prior to treatment and two follow‐up tests during therapy. The patient flow is shown in Figure 1. Patient characteristics are shown in Table 1 separately for CTEPH and for PAH. Patients with PAH were significantly younger and had more severe precapillary pulmonary hypertension.

**Table 1:** Baseline characteristics and treatment at follow‐up.

	CTEPH	PAH	*p*‐value
Baseline characteristics			
*n* (%)	92 (100)	91 (100)	
Idiopathic		49 (53.8)	
Connective tissue disease		25 (27.5)	
Other		17 (18.7)	
Age at diagnosis, years	58 (48–69)	43 (32–58)	< 0.001
Females, *n* (%)	40 (43)	65 (71)	< 0.001
BMI, kg/m^2^	25.8 (22.6–29.1)	23.3 (20.8–29.1)	0.04
mPAP, mmHg	37 (28–43)	46 (31–57)	< 0.01
CO, L/min	5 (4.2–6.2)	4.9 (4.2–6.1)	0.9
CI, L/min/m^2^	2.7 (2.2–3.2)	2.8 (2.3–3.3)	0.3
PVR, WU	5.5 (3.5–8.1)	7 (3.2–10)	0.04
PAWP, mmHg	11 (8.9–14)	10 (9–13)	0.1
DL_CO_, % predicted	67 (61–82)	62 (55–77)	0.06
FVC, % predicted	95 (86–105)	89 (73–100)	0.06
FEV1, % predicted	91 (81–102)	88 (77–101)	0.4
FEV1/FVC, % predicted	95 (90–103)	99 (92–104)	0.2
LVEF, %	61 (56–63)	62 (59–65)	0.2
Treatment at follow‐up			
Pulmonary vasodilators, *n* (%)			
− Phosphodiesterase 5‐inhibitor	17 (18.5)	72 (79)	< 0.001
− Soluble guanylate cyclase stimulator	43 (46.7)	17 (19)	< 0.001
− Endothelin receptor antagonist	53 (57.6)	81 (89)	< 0.001
− Prostaglandins	1 (1.1)	15 (16.5)	< 0.001
− Selective IP prostacyclin receptor agonist	1 (1.1)	23 (25.3)	< 0.001
− Activin signaling inhibitor	0 (0)	12 (13)	< 0.001
No targeted drug therapy	24 (26.1)	2 (2.2)	< 0.001
Monotherapy	31 (33.7)	21 (23.1)	< 0.01
Combination therapy	36 (39.1)	27 (29.7)	0.02
Triple therapy	1 (1.1)	27 (29.7)	< 0.001
Calcium channel blocker	0 (0)	33 (36)	< 0.001
Oral anticoagulant	92 (100)	30 (33)	< 0.001
Balloon pulmonary angioplasty (BPA), *n* (%)	23 (25)	0 (0)	< 0.001
Pulmonary endarterectomy (PEA), *n* (%)	49 (53)	0 (0)	< 0.001
PEA and BPA, *n* (%)	6 (6.5)	0 (0)	< 0.001
PEA and drug therapy, *n* (%)	27 (29.3)	0 (0)	< 0.001

*Note:* Baseline characteristics of patients with chronic thrombo‐embolic pulmonary hypertension (CTEPH) or with pulmonary arterial hypertension (PAH) before treatment. Data are reported as median (25th−75th percentile) or as *n* (%). Comparison between groups with Pearson's chi‐square or Wilcoxon rank sum test. BMI, body‐mass‐index; CO, cardiac output; DLCO, diffusion capacity for carbon monoxide; FVC, forced vital capacity; FEV1, forced expiratory volume in the first second; mPAP, mean pulmonary artery pressure; PVR, pulmonary vascular resistance; PAWP, pulmonary vascular wedge pressure; LVEF, left ventricular ejection fraction.

### 2.2 Comparison Between CTEPH and PAH at Baseline

Although patients with CTEPH had less severe precapillary pulmonary hypertension, they exhibited significantly worse ventilatory efficiency, as indicated by a higher VE/VCO_2_ slope and VE/CO_2_ at baseline (first CPET independent of treatment) compared to those with PAH (Table 2). When only considering those that were completely untreated at baseline (CTEPH *n* = 56, PAH *n* = 28), there was still a significant higher VE/VCO_2_ slope and VE/CO_2_ in CTEPH patients.

**Table 2:** Ventilatory efficiency and other cardiopulmonary exercise test parameters at baseline.

	CTEPH	PAH	*p*‐value
First CPET			
VE/VCO_2_ slope	*n* = 92 39.8 (34.3–47.5)	*n* = 91 35.9 (31–41.5)	< 0.001
VE/VCO_2_ at AT	*n* = 92 44.8 (39.8–51.6)	*n* = 90 39.8 (33.8–46.1)	< 0.001
Peak VO_2_	*n* = 92 15.9 (13–19.1)	*n* = 91 15.9 (12.9–21.2)	0.6
Baseline CPET (untreated)			
VE/VCO_2_ slope	*n* = 56 42.9 (36.8–54)	*n* = 28 36.8 (33.9–43.4)	0.01
VE/VCO_2_ at AT	*n* = 58 46.8 (42.9–53.8)	*n* = 27 40 (36.6–48.7)	0.002
Peak VO_2_	*n* = 64 15.8 (12.1–18.1)	*n* = 31 13.9 (10.6–15.6)	0.02

*Note:* Ventilatory efficiency and other cardiopulmonary exercise test parameters at baseline in all patients and in treatment‐naïve patients in chronic thromboembolic pulmonary hypertension (CTEPH) and pulmonary arterial hypertension (PAH). Values are reported as median (25th to 75th percentile). VE/VCO_2_ slope, slope of ventilatory equivalent for carbon dioxide; VE/VCO_2_ at AT, ventilatory equivalent for carbon dioxide at aerobic threshold; peak VO_2_, maximal oxygen consumption per kilogram.

### 2.3 Predictors of Ventilatory Efficiency

In untreated CTEPH, both PVR (*p* < 0.001, *R* ^2^ = 29.1%, *b* = 5.2 ± 1% [SE]) and mPAP (*p* < 0.01, *R* ^2^ = 14.4, *b* = 1 ± 0.3% (SE) were independent positive predictors of the VE/VCO_2_ slope, and these associations remained significant after adjustment for age. Peak VO_2_ (*p* < 0.001, *R* ^2^ = 27.5%, *b* = −3.5 ± 0.7% [SE]) was an independent negative predictor of the VE/VCO_2_ slope, also after adjustment for age. Resting paO_2_ (*p* = 0.036, *R* ^2^ = 6.6%, *b* = −5.3 ± 2.5% [SE]) was negatively associated with the VE/VCO_2_ slope; however, this association was no longer significant after adjustment for age. DL_CO_ and VD/VT (at AT and at peak exercise) were not significant predictors. However, resting PaCO_2_ was a strong independent predictor of the VE/VCO_2_ slope (*p* < 0.0001, *R* ^2^ = 44.3%, *b* = −37.7 ± 5.7% [SE]) in CTEPH; this was observed both in patients with CTEPH who underwent PEA (*p* < 0.0001, *R* ^2^ = 53.7%, *b* = −42.1 ± 7.1% [SE]) and in those who were not eligible for PEA (*p* = 0.01, *R* ^2^ = 20.1%, *b* = −24.6 ± 9.3% [SE]), with a stronger association in the PEA group.

In patients with PAH, PVR was an independent positive predictor of the VE/VCO_2_ slope (*p* = 0.016, *R* ^2^ = 7.7%, *b* = 1 ± 0.4% [SE]), and this association remained significant after adjustment for age. The regression coefficient (*b*) was higher when only untreated patients were analyzed (*p* = 0.003, *R* ^2^ = 25.7%, *b* = 2 ± 0.7% [SE], *n* = 27). After adjustment for age, mPAP was also an independent positive predictor of the VE/VCO_2_ slope (*p* = 0.028, *R* ^2^ = 4.4%, *b* = 0.4 ± 0.1% [SE]). This association persisted when only untreated patients were considered (*p* = 0.03, *R* ^2^ = 17%, *b* = 0.7 ± 0.3% [SE], *n* = 27). Peak VO_2_ showed a significant negative association with the VE/VCO_2_ slope (*p* < 0.001, *R* ^2^ = 29.1%, *b* = −2 ± 0.03% [SE]), also after correcting for age and when considering untreated patients only (*p* = 0.02, *R* ^2^ = 16.1%, *b* = −2 ± 0.7% [SE], *n* = 27). VD/VT at AT (*p* < 0.001, *R* ^2^ = 14.3%, *b* = 1.4 ± 0.3% [SE]) and at peak exercise (*p* < 0.001, *R* ^2^ = 13.1%, *b* = 1.3 ± 0.4% [SE]) were both positively associated with the VE/VCO_2_ slope, and these associations remained significant after adjustment for age. Baseline resting PaCO_2_ was also a predictor of the VE/VCO_2_ slope at baseline in PAH (*p* < 0.001, *R* ^2^ = 27.4%, *b* = −26.8 ± 8.2% [SE]). No significant association was observed between DL_CO_ or paO_2_ and the VE/VCO_2_ slope.

### 2.4 Effect of Treatment of CTEPH on Ventilatory Efficiency and Gas Exchange

**Figure 2:**
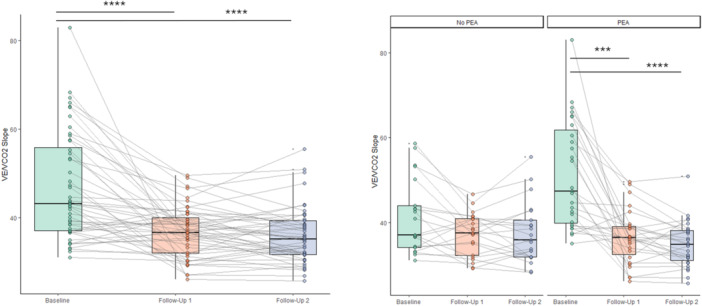
VE/VCO_2_ slope in CTEPH patients and CTEPH patients with no PEA versus PEA. CTEPH, chronic thromboembolic pulmonary hypertension; PEA, pulmonary endarterectomy. *** *p* < 0.0001 and **** *p* < 0.001.

**Figure 3:**
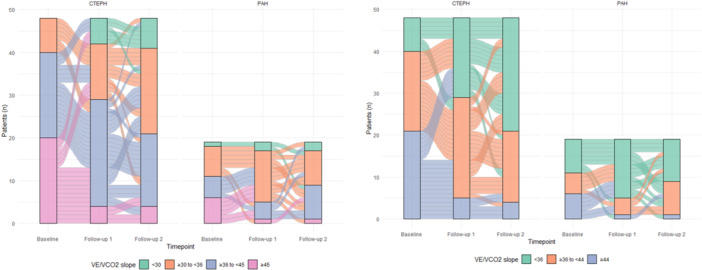
(A) VE/VCO_2_ slope alluvial plot in CTEPH. Cut‐off < 30, ≥ 30 to < 36, ≥ 36 to < 45, ≥ 45. (B) VE/VCO_2_ slope alluvial plot in CTEPH. Cut‐off < 36, ≥ 36 to < 44, ≥ 44. CTEPH, chronic thromboembolic pulmonary hypertension.

Forty‐eight patients with CTEPH had both a baseline CPET before treatment and a follow‐up CPET following treatment. In these, there was a statistically significant improvement in ventilatory efficiency as indicated by a decrease in VE/VCO_2_ slope and VE/VCO_2_ at AT at second follow‐up by −8 (95% CI −13.3 to −4.2, *p* < 0.001) and −6.6 (95% CI −11.5 to −3.7, *p* < 0.001) accompanied by a significant improvement in peak VO_2_ (2.5 [95% CI 1–3.7, *p* < 0.001]) (Tables 3 and 4, Figure 2). Of these 48 patients, 43.8% were in a high‐risk VE/VCO_2_ slope group of which 42.9% changed from a high to an intermediate and 33.3% from a high to a low‐risk VE/VCO_2_ slope group after first follow‐up (Figure 3). Arterial blood gas analysis at rest at baseline demonstrated hypocapnia, that is, respiratory alkalosis, which decreased significantly at follow‐up in the CTEPH group, particularly among patients who underwent PEA (Table 5).

**Table 3:** Change in ventilatory efficiency and other cardiopulmonary exercise test parameters.

	VE/VCO_2_ slope at baseline (untreated)	Delta BL‐ FU1 (95% CI)	VE/VCO_2_ slope at first follow‐up	Delta BL FU2 (95% CI)	VE/VCO_2_ slope at second follow‐up
CTEPH (*n* = 48)	43.2 (37.1–55.8)	−6.5 (−12 to −2.5) *p* < 0.001	36.7 (32.1–40)	−8 (−13.3 to −4.2) *p* < 0.001	35.2 (31.7–39.3)
CTEPH with PEA (*n* = 27)	47.3 (39.8–61.8)	−10.8 (−22.4 to −5.2) *p* < 0.001	36.5 (32.5–39.1)	−12.5 (−24.1 to −6.4) *p* < 0.001	34.8 (31.1–37.1)
CTEPH without PEA (*n* = 21)	37 (34.1–43.9)	0.5 (−5.9 to 3.4) *p* = 0.08	37.5 (32.2–40.9)	−1.1 (−8.8 to 2.4) *p* = 0.06	35.9 (31.9–40.6)
PAH (*n* = 19)	37 (34–48.8)	−2.3 (−11.8 to 0.1) *p* = 0.04	34.7 (32.2–36.8)	−1.4 (−10.9 to 1.9) *p* = 0.06	35.6 (33.9–37.2)

*Note:* Change in ventilatory efficiency. Values are reported as median (25th to 75th percentile and 95% confidence interval). VE/VCO_2_ slope, slope of ventilatory equivalent for carbon dioxide; VE/VCO_2_ at AT, ventilatory equivalent for carbon dioxide at aerobic threshold.

**Table 4:** Change in cardiopulmonary exercise (CPET) parameters over time in patients with three CPETs.

	VE/VCO_2_ BL (untreated)	Delta BL‐FU1	VEVCO_2_ FU1	Delta BL‐FU2	VE/VCO_2_ FU2
CTEPH (*n* = 49)	47.1 (43.1–54.2)	−5 (−9.6 to −2.2)	42.1 (39.3–47.4)	−6.6 (−11.5 to −3.7)	40.5 (36.9–46.1)
CTEPH + PEA (*n* = 26)	51.1 (45.2–64.2)	−8.2 (−16.5 to 2.6)	42.9 (38.3–46.9)	−11.2 (−18.3 to −5)	39.9 (36.8–46)
CTEPH without PEA (*n* = 23)	45 (41–49.8)	−3.6 (−6.1 to 0.5)	41.4 (39.4–47.5)	−4.4 (−7.3 to −0.4)	40.6 (37.3–45.7)
PAH (*n* = 19)	40 (36.6 48.9)	−1.4 (−7.2 to 2)	38.6 (35.6–42.2)	−0.9 (−6.6 to 2.3)	39.1 (38.1–40.6)
	**Peak VO**_**2**_ **BL (untreated)**	**Delta BL‐FU1**	**Peak VO2 FU1**	**Delta BL‐FU2**	**Peak VO2 FU2**
CTEPH (*n* = 58)	15.8 (12.2–18.1)	2.2 (0.3–3.8)	18 (14.2–21.1)	2.5 (1–3.7)	18.3 (13.8–21.5)
CTEPH + PEA (*n* = 34)	14 (11.5–16.9)	2.7 (0.8–5.2)	16.7 (13.8–19.8)	2.6 (0.9–5.1)	16.6 (13.4–20.8)
CTEPH without PEA (*n* = 24)	16.6 (15.8–21.8)	3.3 (−0.2 to 4.7)	19.9 (15.8–22.3)	2.5 (−0.5 to 4.3)	19.1 (16.7–22.6)
PAH (*n* = 21)	13.7 (10.3–15.8)	1.9 (0.1–6.7)	15.6 (13.1–19.2)	2.8 (−0.6 to 6.7)	16.5 (13.1–19.7)

*Note:* Change in CPET parameters. Values are reported as median (25th to 75th percentile and 95% confidence interval). VE/VCO_2_ slope, slope of ventilatory equivalent for carbon dioxide; VE/VCO_2_ at AT, ventilatory equivalent for carbon dioxide at aerobic threshold.

**Table 5:** Arterial blood gas analysis during cardiopulmonary exercise.

	Time	CTEPH	CTEPH with PEA	CTEPH without PEA	PAH
Baseline CPET					
pH	At rest	*n* = 52 7.45 (7.43–7.46)	*n* = 28 7.45 (7.42–7.46)	*n* = 24 7.44 (7.43–7.45)	*n* = 25 7.43 (7.41–7.45)
	At max	*n* = 40 7.40 (7.38–7.44)	*n* = 21 7.40 (7.39–7.44)	*n* = 19 7.39 (7.36–7.43)	*n* = 18 7.39 (7.35–7.42)
paCO_2_	At rest	*n* = 55 4.4 (4.1–4.8)	*n* = 29 4.3 (4.0–4.7)	*n* = 26 4.6 (4.3–4.8)	*n* = 24 4.5 (4.1–4.9)
	At max	*n* = 44 4.2 (3.9–4.5)	*n* = 22 4.2 (3.6–4.5)	*n* = 22 4.2 (4.0–4.4)	*n* = 18 4.2 (3.9–4.6)
paO_2_	At rest	*n* = 55 9.3 (8.6–10.5)	*n* = 29 8.8 (8.4–9.4)	*n* = 26 10.2 (9.2–10.9)	*n* = 25 10.9 (9.7–12.0)
	At max	*n* = 44 8.3 (7.3–9.6)	*n* = 22 7.7 (7.0–9.0)	*n* = 22 9.1 (7.9–9.8)	*n* = 18 11.2 (8.2–11.5)
SaO_2_	At rest	*n* = 51 95 (93–96)	*n* = 28 93.4 (92.8–95)	*n* = 23 96 (95–96.9)	*n* = 25 97 (96–98)
	At max	*n* = 39 92 (89–95)	*n* = 21 90 (87–93)	*n* = 18 94.3 (92–96.07)	*n* = 17 95 (90–96.7)
HCO_3_^−^	At rest	*n* = 51 24.1 (22.8–25.1)	*n* = 28 23.9 (22.5–24.7)	*n* = 23 24.5 (23.2–25.3)	*n* = 22 23 (22.4–24.5)
	At max	*n* = 39 20.9 (19.9–21.5)	*n* = 21 20.9 (19.8–21.3)	*n* = 18 20.9 (20.1–21.7)	*n* = 17 20.4 (18.4–22.1)
CPET first follow‐up					
pH	At rest	*n* = 82 7.44 (7.42–7.47)	*n* = 43 7.43 (7.42–7.44)	*n* = 39 7.44 (7.43–7.47)	*n* = 71 7.45 (7.42–7.46)
	At max	*n* = 64 7.37 (7.35–7.40)	*n* = 31 7.38 (7.34–7.40)	*n* = 33 7.37 (7.35–7.40)	*n* = 47 7.40 (7.36–7.43)
paCO_2_	At rest	*n* = 84 4.6 (4.3–4.9)	*n* = 45 4.6 (4.3–5.0)	*n* = 39 4.5 (4.1–4.9)	*n* = 72 4.7 (4.4–5.0)
	At max	*n* = 66 4.5 (4.1–4.9)	*n* = 33 4.4 (4.2–4.9)	*n* = 33 4.5 (4.1–4.8)	*n* = 48 4.4 (3.9–4.8)
paO_2_	At rest	*n* = 84 10.1 (9.2–11.0)	*n* = 45 10.3 (9.3–11)	*n* = 39 9.9 (9.1–10.9)	*n* = 73 10.5 (9.6–11.4)
	At max	*n* = 66 8.4 (7.7–9.9)	*n* = 33 9.0 (7.4–9.9)	*n* = 33 8.2 (7.7–9.6)	*n* = 48 9.5 (8.1–11.0)
SaO_2_	At rest	*n* = 82 96 (94–97)	*n* = 43 96 (94.4–97)	*n* = 39 96 (94–97)	*n* = 71 97 (95–97)
	At max	*n* = 64 92 (89.8–95)	*n* = 31 93 (89.5–95.5)	*n* = 33 91.5 (90–95)	*n* = 48 94.5 (92–96)
HCO_3_^−^	At rest	*n* = 82 24.2 (23.1–25.1)	*n* = 44 24.1 (23.0–25.2)	*n* = 38 24.3 (23.4–25.0)	*n* = 70 24.7 (23.1–25.8)
	At max	*n* = 65 20.5 (19.4–21.5)	*n* = 32 20.5 (19.2–22.2)	*n* = 33 20.5 (19.7–21.1)	*n* = 46 21.3 (19.4–22.7)
CPET second follow‐up					
pH	At rest	*n* = 84 7.44 (7.43–7.47)	*n* = 44 7.44 (7.42–7.46)	*n* = 40 7.45 (7.44–7.47)	*n* = 76 7.44 (7.42–7.46)
	At max	*n* = 71 7.38 (7.36–7.41)	*n* = 33 7.38 (7.35–7.41)	*n* = 38 7.38 (7.36–7.40)	*n* = 64 7.39 (7.34–7.42)
paCO_2_	At rest	*n* = 84 4.6 (4.4–4.9)	*n* = 44 4.6 (4.4–5.0)	*n* = 40 4.7 (4.1–4.9)	*n* = 76 4.7 (4.4–5.0)
	At max	*n* = 71 4.5 (4.1–4.9)	*n* = 33 4.6 (4.1–4.9)	*n* = 38 4.5 (4.1–4.8)	*n* = 64 4.5 (4.1–4.9)
paO_2_	At rest	*n* = 84 10.3 (9.1–11.1)	*n* = 44 10.5 (9.4–11.4)	*n* = 40 10.0 (9.1–10.6)	*n* = 76 10.4 (9.4–11.5)
	At max	*n* = 71 8.5 (7.7–10.0)	*n* = 33 9.1 (8.0–10.0)	*n* = 38 8.3 (7.7–9.8)	*n* = 64 9.4 (7.9–10.6)
SaO_2_	At rest	*n* = 84 96 (95–97)	*n* = 44 96 (95–97)	*n* = 40 96 (95–97)	*n* = 76 96 (94.5–97)
	At max	*n* = 69 93 (90–95)	*n* = 31 94 (90.9–95)	*n* = 38 92 (89–95)	*n* = 64 93.5 (90–96.1)
HCO_3_^‐^	At rest	*n* = 84 24.6 (23.6–25.3)	*n* = 44 24.4 (23.6–25.2)	*n* = 40 24.7 (23.9–25.4)	*n* = 76 24.4 (23.4–25.7)
	At max	*n* = 69 20.9 (19.6–22.0)	*n* = 31 20.7 (19.55–22.5)	*n* = 38 21.0 (19.8–21.7)	*n* = 63 21.3 (19.9–22.6)

*Note:* Arterial blood gases. Values are reported as median (25th to 75th percentile and 95% confidence interval). CTEPH, chronic thromboembolic pulmonary hypertension; PAH, pulmonary arterial hypertension; PEA, pulmonary endarterectomy.

### 2.5 Effect of Treatment of PAH on Ventilatory Efficiency and Gas Exchange

**Figure 4:**
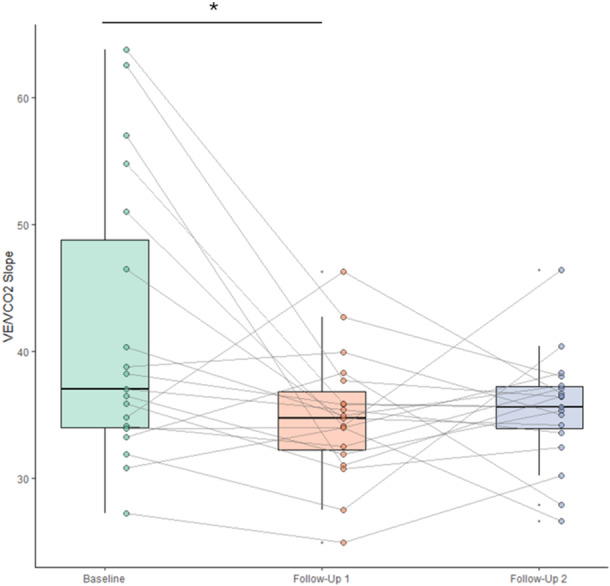
VE/VCO_2_ slope in PAH. *p < 0.05. PAH, pulmonary arterial hypertension.

Nineteen patients with PAH had a baseline CPET before treatment and a follow‐up CPET following treatment. In these, there was only a mild improvement in ventilatory efficiency in response to treatment initiation (Tables 3 and 4, Figure 4), while some individual patients deteriorated and improved at second follow‐up due to extension of therapy.

## 3 Discussion

In this observational study, we compared ventilatory efficiency between patients CTEPH and PAH and examined its evolution over time in response to treatment. Several important findings emerged. First, despite less severe hemodynamic impairment, patients with CTEPH exhibited significantly worse ventilatory efficiency at baseline than those with PAH. Second, ventilatory inefficiency in both conditions was associated with hemodynamic severity and exercise capacity, although the physiological determinants differed partially between the two groups. Third, treatment was associated with a marked improvement in ventilatory efficiency in CTEPH, particularly following PEA, whereas only modest changes were observed in PAH after initiation or escalation of targeted therapy.

At baseline, patients with CTEPH demonstrated significantly higher VE/VCO_2_ slope and VE/VCO_2_ at the anaerobic threshold compared with patients with PAH. Importantly, this difference persisted when the analysis was restricted to treatment‐naïve patients, suggesting that the observed disparity is explained by the underlying pathophysiology rather than related to treatment. Notably, this occurred despite patients with PAH presenting with higher baseline mPAP and pulmonary vascular resistance. Previous comparative studies with similar resting hemodynamics in CTEPH and PAH support the cross‐sectional observation that ventilatory efficiency is more impaired in CTEPH than in PAH [18, 19]. These findings may reflect differences in pulmonary vascular pathophysiology between the two diseases. In CTEPH, persistent organized thromboembolic obstruction leads to marked perfusion heterogeneity and an increased proportion of ventilated but poorly perfused lung units. This results in an increased physiological dead space and an exaggerated ventilatory response during exercise. In contrast, PAH is characterized predominantly by diffuse small‐vessel vasculopathy, which tends to produce a more homogeneous distribution of perfusion abnormalities. Although hemodynamic impairment may be more severe in PAH, the degree of ventilation–perfusion mismatch may be less pronounced than in CTEPH, potentially explaining the relatively better ventilatory efficiency in PAH.

Consistent with previous studies, hemodynamic severity was an important determinant of ventilatory inefficiency [20, 21]. In both CTEPH and PAH, pulmonary vascular resistance and mPAP were independent positive predictors of the VE/VCO_2_ slope, while peak oxygen uptake was inversely associated with ventilatory inefficiency. Therefore, the VE/VCO_2_ slope may represent a useful non‐invasive parameter for the detection of precapillary pulmonary hypertension or exercise‐induced pulmonary hypertension in at‐risk populations, including patients with systemic sclerosis [13].

Of interest, in CTEPH the VE/VCO_2_ slope was not predicted by VD/VT at anaerobic threshold, whereas resting PaCO_2_ showed a significant inverse association with the VE/VCO_2_ slope (strongest association in those undergoing PEA). This suggests that ventilatory inefficiency in CTEPH may be driven more by heightened ventilatory drive, with partial normalization after PEA. Whereas in PAH both VD/VT and resting PaCO_2_ were significant predictors of the VE/VCO_2_ slope, in CTEPH only PaCO_2_ showed a significant association with the slope; moreover, the relationship between hypocapnia and the VE/VCO_2_ slope was stronger in CTEPH, and strongest in patients who underwent PEA.

Treatment of CTEPH was associated with a substantial improvement in ventilatory efficiency, particularly in patients undergoing PEA (VE/VCO_2_ slope −26.4%) of which 55% were also on targeted drug therapy. Previous studies have similarly demonstrated that effective treatment of CTEPH improves ventilatory efficiency, with PEA yielding improvements of a comparable magnitude (approximately 21%–26%) and with greater improvements observed in patients with poorer baseline ventilatory efficiency [22, 23, 24]. In 20 patients treated with PEA, VE/VCO_2_ decreased by 26% from 50 ± 9 to 37 ± 5, accompanied by marked improvements in hemodynamics, including reductions in mPAP and pulmonary vascular resistance [23]. In another study of 38 patients undergoing BPA (mean 4.5 ± 1.8 sessions) in combination with pulmonary vasodilator therapy, peak exercise VE/VCO_2_ decreased significantly by 17% from 57.8 ± 16.7 to 48.0 ± 12.6 (*p* < 0.001), which is comparable to the reduction observed in our BPA patients (−15%) [25]. The magnitude of improvement observed in our cohort is clinically relevant. A considerable proportion of patients moved from a high‐risk VE/VCO_2_ slope category to intermediate‐ or low‐risk categories following treatment. This finding highlights the potential of CPET to capture physiological improvements after successful intervention and suggests that ventilatory efficiency may represent a sensitive marker of treatment response in CTEPH.

In contrast to CTEPH, only modest changes in ventilatory efficiency were observed in patients with PAH following initiation or escalation of targeted medical therapy. Although small improvements in VE/VCO_2_ slope were seen at first follow‐up, these changes were limited and not consistently sustained at later follow‐up assessments. Individual patient trajectories varied, with some patients improving and others deteriorating over time, likely reflecting the heterogeneous response to pharmacological therapy and the progressive nature of the disease.

Ventilatory inefficiency has also been shown to carry important prognostic information in pulmonary vascular disease. In a cohort of 116 patients with PAH (mean mPAP 35 ± 1 mmHg), an increased VE/VCO_2_ slope was a significant predictor of 24‐month mortality, underscoring the value of ventilatory efficiency as a risk stratification parameter [12, 13]. Given its strong predictive value, an elevated VE/VCO_2_ should prompt treatment escalation and, in cases of maximal therapy, eventually consideration of referral for lung transplant evaluation.

Our findings reinforce the importance of ventilatory efficiency as a physiological marker in pulmonary vascular disease. Ventilatory efficiency may be particularly useful for monitoring treatment response and functional recovery following therapies. Furthermore, the observation that patients with CTEPH exhibit worse ventilatory efficiency despite less severe hemodynamic impairment highlights the limitations of resting hemodynamic measurements alone in capturing disease burden.

Several limitations should be considered when interpreting these findings. First, this was a retrospective analysis from a single center, which may limit generalizability. Second, the number of patients with serial cardiopulmonary exercise tests was relatively limited, particularly in the PAH group, which may have reduced statistical power to detect treatment‐related changes. Third, the heterogeneity of treatments, especially in PAH, may have contributed to variability in longitudinal responses. In addition, the two groups differed in age, sex, BMI, and hemodynamics, precluding a direct comparison of treatment effects between PAH and CTEPH.

## 4 Conclusions

Patients with CTEPH exhibit significantly worse ventilatory efficiency than patients with PAH despite less severe hemodynamic impairment, likely reflecting greater ventilation‐perfusion mismatch. Ventilatory inefficiency in both diseases is associated with hemodynamic severity and exercise capacity. Treatment results in substantial improvements in ventilatory efficiency in CTEPH, particularly following PEA, whereas changes are more modest in PAH. These findings highlight the value of CPET as non‐invasive tool in characterizing disease physiology and monitoring treatment response.

## Author Contributions

Conception of the work: Esther I. Schwarz. Analysis: Lara Benning. Interpretation of data for the work: All. Drafting the work: Esther I. Schwarz, Lara Benning. Reviewing it critically for important intellectual content and final approval of the version to be published: All.

## Funding

The authors have nothing to report.

## Ethics Statement

The study was approved by the Ethics Committee of the Kanton of Zurich, Switzerland: KEK 2025‐02640.

## Conflicts of Interest

The authors declare no conflicts of interest. Esther I. Schwarz reports funding from Löwenstein and SNF outside of the submitted work. Silvia Ulrich reports support from Janssen SA, Swiss Lung League, Orpha Swiss, MSD Switzerland, Gebro Swiss and SNF outside the submitted work. Isabelle Opitz reports funding outside the submitted work from Roche (Institutional Grant), Medtronic (Institutional Grant and Advisory Board) and XVIVO (Institutional Grant).

## Data Availability

The data that support the findings of this study are available from the corresponding author upon reasonable request.
